# Impact of renal function on patients with acute coronary syndromes: 15,593 patient-years study

**DOI:** 10.1080/0886022X.2020.1810069

**Published:** 2020-08-30

**Authors:** Łukasz Kuźma, Jolanta Małyszko, Anna Kurasz, Marta Maria Niwińska, Małgorzata Zalewska-Adamiec, Hanna Bachórzewska-Gajewska, Sławomir Dobrzycki

**Affiliations:** a Department of Invasive Cardiology, Medical University of Bialystok, Bialystok, Poland; b Department of Clinical Medicine, Medical University of Bialystok, Bialystok, Poland

**Keywords:** Chronic kidney disease, acute coronary syndrome, STEMI, NSTEMI, contrast-induced acute kidney disease, glomerular filtration rate, contrast-induced nephropathy

## Abstract

**Introduction:**

Coexistence of chronic kidney disease (CKD) in the case of acute coronary syndromes (ACS) significantly worsens the outcomes.

**Aim:**

The aim of our study was to assess renal function impact on mortality among patients with ACS.

**Materials and methods:**

The study was based on records of 21,985 patients hospitalized in the Medical University of Bialystok in 2009–2015. Inclusion criteria were ACS. Exclusion criteria were: death within 24 h of admission, eGFR <15 ml/min/1.73 m^2^, hemodialysis. Mean observation time was 2296 days.

**Results:**

Criteria were met by 2213 patients. CKD occurred in 24.1% (*N* = 533) and more often affected those with NSTEMI (26.2 (337) vs. 21.2 (196), *p* = .006). STEMI patients had higher incidence of post-contrast acute kidney injury (PC-AKI) (5 (46) vs. 4.1 (53), *p* < .001). During the study, 705 people died (31.9%), more often with NSTEMI (33.2% (428) vs. 29.95% (277), *p* < .001). However, from a group of patients suffering from PC-AKI 57.6% died. The risk of PC-AKI increased with creatinine concentration (RR: 2.990, 95%CI: 1.567–5.721, *p* < .001), occurrence of diabetes mellitus (RR: 2.143, 95%CI: 1.029–4.463, *p* = .042), atrial fibrillation (RR: 2.289, 95%CI: 1.056–4.959, *p* = .036). Risk of death was greater with an increase in postprocedural creatinine concentration (RR: 2.254, 95%CI: 1.481–3.424, *p* < .001).

**Conclusion:**

PC-AKI is a major complication in patients with ACS, occurs more frequently in STEMI and may be a prognostic marker of long-term mortality in patients undergoing percutaneous coronary intervention (PCI). More attention should be given to the prevention and diagnosis of PC-AKI but necessary PCI should not be withheld in fear of PC-AKI.

## 1. Background

Cardiovascular diseases are the leading cause of mortality among the Polish population. These are responsible for 46% of deaths – it is almost twice as much as deaths caused by cancer (27%) [1]. Kidney disease affects cardiac function and can change the course and prognosis of patients with acute coronary syndromes (ACS) [2].

A Polish study on the assessment of the incidence of chronic kidney disease (CKD) among the Polish population claims that CKD affects about 5.8%, where in the case of seniors the percentage is 26.9%. Some studies suggest that CKD is the greatest risk factor for cardiovascular diseases occurrence [3–5].

CKD is highly prevalent worldwide and is associated with an increased risk for adverse outcomes in patients hospitalized due to ACS, the co-occurrence of these two diseases increases the risk of death [6].

The gold standard of treatment for patients with ACS is percutaneous coronary intervention (PCI). Many patients after PCI experience a decrease in GFR, which is a certain group manifests itself as post-contrast acute kidney injury (PC-AKI).

Data on the impact of post-procedural eGFR decline on mortality in patients with ACS are still limited – it is known that this condition is associated with more adverse outcomes [7].

Therefore, in our study, we aimed to investigate which factors predispose to contrast-induced acute kidney injury and how it affects mortality among patients with ST-segment elevation myocardial infarction (STEMI) and non-ST-segment elevation myocardial infarction (NSTEMI).

## 2. Patients and methods

The study was conducted at the Clinical Hospital of Medical University of Bialystok. Based on the medical records of 2258 men and women hospitalized due to ACSs in 2009 − 2015.

The demographic, clinical, and biochemical data of the patients were evaluated. The diagnosis of STEMI and NSTEMI was made by physicians based on the symptoms, level of biochemical markers of myocardial necrosis, and electrocardiographic results.

All patients underwent coronary angiography. During all procedures, an iodine-containing nonionic radiocontrast agent was used. All patients had the same strategy for the prevention of radiocontrast agent – 1000 mL of intravenous hydration with 0.9% NaCl.

CKD-EPI eGFR and creatinine levels were assessed on admission and 48 h after angiography, based on age, gender, race, and creatinine concentration [8]. For the purpose of this study, PC-AKI was defined as an absolute increase of serum creatinine ≥0.5 mg/dL or relative increase ≥25% from the baseline value within the first 48 h of intervention. Currently, newer, more strict criteria are available [9], however, given that the study covers patients hospitalized between 2009 and 2015, thus the end of the study took place before the new guidelines appeared, we decided to use values current for this period.

Around 1.19% of the data were missing during the study period, and these data were excluded from the analysis.

The study protocol was approved by the ethics committees of the Medical University of Bialystok (R-1-002/18/2019).

### Exclusion criteria

Exclusion criteria were: death within 24 h of admission, eGFR <15 mL/min, and hemodialysis. Total 1.9% (*N* = 45) of patients were excluded from the study, in majority male (60%, *N* = 27), 54% (*N* = 25) hospitalized with STEMI, mean eGFR 31.3 mL/min (SD = 26), median = 21.2 mL/min of which 22.2% (*N* = 10) were on hemodialysis.

In this group, the main causes of death were cardiovascular diseases 93.3% (*N* = 42) of which 51.1% of death was reported in 24 h of admission (*N* = 23).

### Long-term observation

The data concerning the cause of deaths that were recorded on April 1, 2019, were obtained from the Statistical Office in Olsztyn, Poland. The records included the causes of deaths that were classified according to codes in the International Classification of Diseases – 10th Revision (ICD-10).

The mean follow-up period from the onset to the death was 1319 (SD = 1070), median – 1230 days. The complete follow-up duration consisted of 2296 days (SD = 1081), median – 2359.

### Statistical analysis

The distribution of variables was evaluated using the Kolmogorov–Smirnov test. The two-tailed *t*-test and Mann–Whitney *U* test were used for comparative analysis. Spearman’s rank correlation test was applied to evaluate the relationships between the levels of postprocedural decrease of eGFR and biochemical parameters.

The effects of clinical and biochemical factors on death were evaluated by multivariable logistic regression backward stepwise Wald method.

Associations were considered significant at *p* < .05. All analyses were performed using Statistica 13 software (StatSoft, 2017, Poland).

## 3. Results

**Figure 1.**
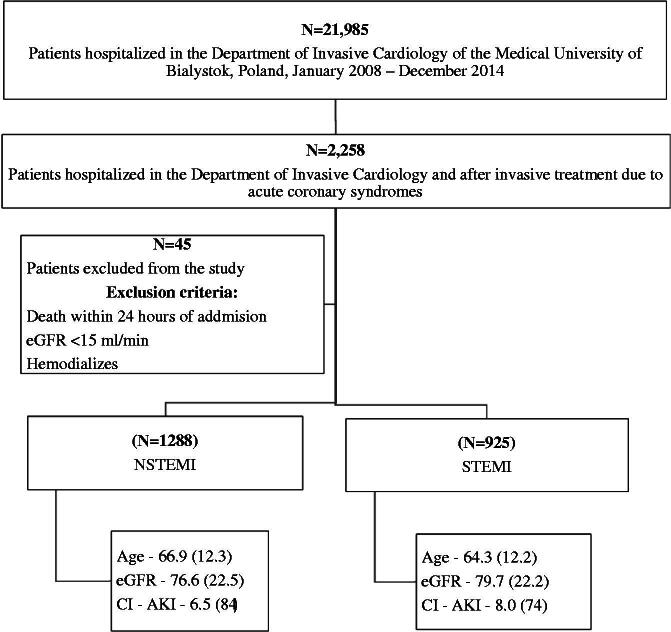
Selection of study population.

A total of 2213 patients were included into the final analysis – 1288 with NSTEMI and 925 with STEMI. The average age was 65.8 years (SD = 12.2) and men were in the majority (65.2%, *N* = 1442) (Figure 1).

Comparison of patients with NSTEMI and those with STEMI shows statistically significant differences. The NSTEMI group was older (66.9 (SD = 12.1) vs. 64.3 (SD = 12.2), *p* < .001), had lower serum LDL (129.7 (SD = 45.1) vs. 140.1 (SD = 44.8), *p* < .001) and total serum cholesterol concentration (198.4 (SD = 48.9) vs. 207.9 (48.9), *p* < .001). They were more often burdened with arterial hypertension (77.0% (*N* = 989) vs. 66.0% (*N* = 606), *p* < .001) and diabetes mellitus type 2 (26.4% (*N* = 340) vs. 18.7% (*N* = 173), *p* < .001). The average eGFR was greater in patients with STEMI (79.7 (SD = 20.8) vs. 76.61 (SD = 21.5), *p* < .001), they also had higher incidence of post-contrast acute kidney injury (5% (*N* = 46) vs. 4.1% (*N* = 53), *p* < .001) (Table 1).

**Table 1.** Characteristics of the studied population with comparison of patients with STEMI and NSTEMI.

Parameter	General population (*N* = 2213)	NSTEMI (*N* = 1288)	STEMI (*N* = 925)	*p*
Age (years), mean, (SD)	65.8 (12.2)	66.9 (12.1)	64.3 (12.2)	<.001
Male, % (*N*)	65.2 (1442)	63.7 (821)	67.1 (621)	.097
BMI (kg/m^2^), mean, (SD)	30.0 (24.8)	30.1 (26.0)	29.7 (22.9)	.687
Arterial hypertension, % (*N*)	72.0 (1595)	77.0 (989)	66.0 (606)	<.001
Diabetes mellitus type 2, % (*N*)	23.2 (513)	26.4 (340)	18.7 (173)	<.001
Atrial fibrillation, % (*N*)	18.0 (398)	17.8 (229)	18.3 (169)	.767
Chronic kidney disease, % (*N*)	24.1 (533)	26.2 (337)	21.2 (196)	.006
Serum creatinine concentration (mg/dl), mean, (SD)	1.0 (0.3)	1.0 (0.3)	1.0 (0.3)	.092
Serum creatinine concentration after 48 h (mg/dl), mean, (SD)	1.0 (0.6)	1.0 (0.4)	1.04 (0.5)	.18
CI - AKI, % (*N*)	4.5 (99)	4.1 (53)	5.0 (46)	<.001
eGFR ml/min/1.73 m², mean, (SD)	77.9 (21.3)	76.6 (21.5)	79.7 (20.8)	<.001
Patients with eGFR ≥90 ml/min/1.73 m², % (*N*)	33.5 (742)	32.4 (417)	35.1 (325)	.177
Patients with eGFR 60–89 ml/min/1.73 m², % (*N*)	45.1 (999)	44.0 (567)	46.7 (432)	.212
Patients with eGFR 45–59 ml/min/1.73 m², % (*N*)	12.6 (279)	14.1 (181)	10.6 (98)	.014
Patients with eGFR 30–44 ml/min/1.73 m², % (*N*)	5.9 (130)	6.5 (84)	5.0 (46)	.119
Patients with eGFR 15–29 ml/min/1.73 m², % (*N*)	2.5 (63)	3.0 (39)	2.6 (24)	.541
Hemoglobin level (g/dl), mean, (SD)	13.8 (1.6)	13.6 (1.6)	13.8 (1.7)	.016
Platelets count (×10^3^/mm^3^), mean, (SD)	225.6 (72.3)	222.0 (69.2)	230.7 (76.1)	.006
Serum uric acid concentration (mg/dl), mean, (SD)	5.9 (3.5)	6.1 (4.4)	5.8 (1.7)	.017
AspAT activity (IU/l), mean, (SD)	70.5 (119.9)	66.2 (123.5)	76.5 (114.5)	.049
ALAT activity (IU/l), mean, (SD)	44.6 (139.3)	39.8 (68.2)	51.1 (198.9)	.111
Cholesterol concentration (mg/dl), mean, (SD)	202.4 (49.2)	198.4 (48.9)	207.9 (48.9)	<.001
LDL concentration (mg/dl), mean, (SD)	134.1 (45.2)	129.7 (45.1)	140.1 (44.8)	<.001
HDL concentration (mg/dl), mean, (SD)	47.3 (13.3)	46.9 (13.6)	47.8 (12.9)	.155
Triglycerides concentration (mg/dl), mean, (SD)	119.4 (85.9)	122.7 (88.6)	114.8 (81.8)	.013
Fibrinogen concentration (mg/dl), mean, (SD)	431.6 (114.0)	434.5 (115.1)	427.6 (114.8)	.388
Death, % (*N*)	31.9 (705)	33.2 (428)	30.0 (277)	<.001

ALAT: alanine aminotransferase; AKI: contrast-induced acute kidney injury; AspAT: aspartate aminotransferase; BMI: body mass index; CI: eGFR: estimated glomerular filtration rate; HDL: high density lipoprotein; LDL: low density lipoprotein; NSTEMI: non-ST segment elevation myocardial infarction; STEMI: ST-segment elevation myocardial infarction.

The characteristic of the population is presented depending on the presence (*N* = 533) and absence (*N* = 1680) of CKD. Patients with CKD more often had comorbidities, such as hypertension (79.7%, *N* = 425), diabetes mellitus type 2 (40.7%, *N* = 217), atrial fibrillation (33.6%, *N* = 179), and they were more likely to die (56.3% (*N* = 300) vs. 24.1% (*N* = 405), *p* < .001). CKD patients have experienced PC-AKI far more often (8.4% (*N* = 45) vs. 3.2% (*N* = 54), *p* < .001) (Table 2).

**Table 2.** Comparison of patients with and without chronic kidney disease.

Parameter	Population with CKD (*N* = 533)	Population without CKD (*N* = 1680)	*p*
Age (years), mean, (SD)	75.3 (8.9)	62.8 (11.6)	<.001
Male, % (*N*)	50.1 (267)	69.9 (1175)	<.001
BMI (kg/m^2^), mean, (SD)	29.1 (12.7)	30.2 (27.2)	.496
Arterial hypertension, % (*N*)	79.7 (425)	69.6 (1170)	<.001
Diabetes mellitus type 2, % (*N*)	40.7 (217)	17.6 (296)	<.001
Atrial fibrillation, % (*N*)	33.6 (179)	13.0 (219)	<.001
Serum creatinine concentration (mg/dl), mean, (SD)	1.4 (0.4)	0.9 (0.1)	<.001
Serum creatinine concentration after 48 h (mg/dl), mean, (SD)	1.5 (0.7)	0.9 (0.2)	<.001
CI–AKI, % (*N*)	8.4 (45)	3.2 (54)	<.001
Hemoglobin level (g/dl), mean, (SD)	12.9 (1.9)	13.9 (1.5)	<.001
Platelets account (×10^3^/mm^3^), mean, (SD)	223 (85.8)	226.5 (67.4)	.091
Serum uric acid concentration (mg/dl), mean, (SD)	7.4 (5.1)	5.5 (2.8)	<.001
AspAT activity (IU/l), mean, (SD)	88.1 (135.6)	65.1 (113.9)	<.001
ALAT activity (IU/l), mean, (SD)	53.9 (124.0)	41.6 (143.7)	.495
Total serum cholesterol concentration (mg/dl), mean, (SD)	188.3 (48.3)	206.7 (48.7)	<.001
Serum LDL cholesterol concentration (mg/dl), mean, (SD)	122.4 (43.2)	137.6 (45.3)	<.001
Serum HDL cholesterol concentration (mg/dl), mean, (SD)	44.7 (13.5)	48.1 (13.1)	<.001
Serum triglyceride concentration (mg/dl), mean, (SD)	115.7 (69.8)	120.5 (90.2)	.272
Fibrinogen concentration (mg/dl), mean, (SD)	463.2 (126.3)	421.8 (109.4)	<.001
Death, % (N)	56.3 (300)	24.1 (405)	<.001

ALAT: alanine aminotransferase; AspAT: aspartate aminotransferase; BMI: body mass index; CKD: chronic kidney disease; CI–AKI, contrast-induced acute kidney injury; HDL: high density lipoprotein; LDL: low density lipoprotein.

**Table 3.** Comparison of patients with and without contrast-induced acute kidney injury.

Parameter	Population with PC–AKI (*N* = 99)	Population without PC–AKI (*N* = 2114)	*p*
Age (years), mean, (SD)	71.1 (11.1)	65.6 (12.2)	<.001
Male, % (N)	65.7 (65)	65.1 (1377)	.920
BMI (kg/m2), mean, (SD)	28.9 (5.0)	28.2 (4.8)	.240
Arterial hypertension, % (*N*)	72.7 (72)	72.00 (1523)	.880
Diabetes mellitus type 2, % (*N*)	38.4 (38)	22.5 (475)	.002
Atrial fibrillation, % (*N*)	35.4 (35)	17.2 (363)	<.001
Chronic kidney disease, % (*N*)	45.5 (45)	23.1 (488)	<.001
Serum creatinine concentration (mg/dl), mean, (SD)	1.2 (0.6)	1.00 (0.3)	<.001
eGFR ml/min/1.73 m², mean, (SD)	65.0 (25.7)	78.5 (20.8)	<.001
Patients with eGFR ≥90 ml/min/1.73 m², % (*N*)	20.2 (20)	34.2 (722)	<.001
Patients with eGFR 60–89 ml/min/1.73 m², % (*N*)	39.4 (39)	45.4 (960)	.230
Patients with eGFR 45–59 ml/min/1.73 m², % (*N*)	15.2 (15)	12.5 (264)	.460
Patients with eGFR 30–44 ml/min/1.73 m², % (*N*)	11.1 (11)	5.6 (119)	.090
Patients with eGFR 15–29 ml/min/1.73 m², % (*N*)	14.1 (14)	2.3 (49)	<.001
Hemoglobin level (g/dl), mean, (SD)	13.1 (2.2)	13.7 (1.6)	.004
Platelets account (×10^3^/mm3), mean, (SD)	217.3 (79)	226 (71.9)	.280
Serum uric acid concentration (mg/dl), mean, (SD)	6.6 (2.4)	5.9 (3.6)	.020
AspAT activity (IU/l), mean, (SD)	172.5 (375.1)	65.7 (88.9)	.007
ALAT activity (IU/l), mean, (SD)	104 (270.7)	41.6 (128.7)	.030
Total serum cholesterol concentration (mg/dl), mean, (SD)	193.6 (51.2)	202.8 (49.1)	.090
Serum LDL cholesterol concentration (mg/dl), mean, (SD)	128.5 (46.1)	134.3 (45.2)	.230
Serum HDL cholesterol concentration (mg/dl), mean, (SD)	47.3 (14.1)	47.3 (13.3)	.880
Serum triglyceride concentration (mg/dl), mean, (SD)	106.2 (57.2)	120.01 (87)	.03
Fibrinogen concentration (mg/dl), mean, (SD)	453.4 (130.3)	430.6 (114.1)	.10
Death, % (*N*)	57.6 (57)	30.7 (648)	<.001

ALAT: alanine aminotransferase; AspAT: aspartate aminotransferase; BMI: body mass index; CKD: chronic kidney disease; eGFR: estimated glomerular filtration rate; HDL: high-density lipoprotein; LDL: low-density lipoprotein; PC–AKI: post-contrast acute kidney injury.

PC-AKI occurred in 4.5% of patients. This group was statistically older (71.1 (SD = 11.1) vs. 65.6 (SD = 12.2), *p* < .001), had higher serum creatinine concentration (1.2 (SD = 0.6) vs. 1.0 (SD = 0.3), *p* < .001), and lower average eGFR (65.6 (SD = 25.7) vs. 78.5 (SD = 20.8), *p* < .001) (Table 3). They also more often suffered from atrial fibrillation (35.4% (*N* = 35) vs. 17.2% (*N* = 363), *p* < .001), CKD (45.5% (*N* = 45) vs. 23.1% (*N* = 488), *p* < .001), diabetes mellitus (38.4% (*N* = 38) vs. 22.5% (*N* = 475), *p* = .002), and also died more frequently (57.6% (*N* = 57) vs. 30.7% (*N* = 648), *p* < .001).

During the study, 705 people died, more often with NSTEMI (33.2% (428) vs. 29.95% (277), *p* < .001) of whom 349 (49.5%) died from cardiovascular reasons and 112 from oncological causes (15.9%). In the NSTEMI group, the percentage of cancer deaths was 17.0% (*N* = 73), cardiovascular deaths were 49.3% (*N* = 216) vs. 14.01% (*N* = 39), and 48.1% (*N* = 133) in the STEMI group. Moreover, 57.6% of patients suffering from PC-AKI died – eight of them died from cancer, 40 due to cardiovascular causes, and two from complications of diabetes. Three deaths, due to acute renal failure, were reported in patients who have not post angiography increase in creatinine concentration. When comparing dead and living patients we can notice that significant differences were found in comorbidities such as diabetes mellitus type 2 (32.2% (*N* = 227) vs. 19.0% (*N* = 286), *p* < .001) and atrial fibrillation (28.9% (*N* = 204) vs. 12.9% (*N* = 194), *p* < .001). The percentage of CKD occurrence was 42.6% (*N* = 300) among the deceased population and it had a significant impact on mortality (*p* < .001) (Table 4).

**Table 4.** Comparison of dead and alive patients.

Parameter	Dead (*N* = 705)	Alive (*N* = 1508)	*p*
Age (years), mean, (SD)	72.4 (10.8)	62.7 (11.6)	<.001
Male, % (N)	62.6 (441)	66.4 (1001)	.081
BMI (kg/m^2^), mean, (SD)	31.7 (37.7)	29.2 (17.1)	.159
NSTEMI, % (*N*)	60.7 (428)	57.0 (860)	.101
STEMI, % (*N*)	39.3 (277)	43.0 (648)	.101
Arterial hypertension, % (*N*)	74.9 (528)	70.8 (1067)	.040
Diabetes mellitus type 2, % (*N*)	32.2 (227)	19.0 (286)	<.001
Atrial fibrillation, % (*N*)	28.9 (204)	12.9 (194)	<.001
Chronic kidney disease, % (*N*)	42.6 (300)	15.5 (233)	<.001
Serum creatinine concentration (mg/dl), mean, (SD)	1.1 (0.4)	0.9 (0.3)	<.001
Serum creatinine concentration after 48 hours (mg/dl), mean, (SD)	1.2 (0.6)	1.0 (0.3)	<.001
PC- AKI, % (*N*)	8.1 (57)	2.78 (42)	<.001
eGFR ml/min/1.73 m², mean, (SD)	67.1 (22.6)	82.9 (18.5)	<.001
Patients with eGFR ≥90 ml/min/1.73 m², % (*N*)	16.6 (117)	41.4 (625)	<.001
Patients with eGFR 60–89 ml/min/1.73 m², % (*N*)	44.8 (316)	45.3 (683)	.836
Patients with eGFR 45–59 ml/min/1.73 m², % (*N*)	19.7 (139)	9.3 (140)	<.001
Patients with eGFR 30–44 ml/min/1.73 m², % (*N*)	12.1 (85)	3.0 (45)	<.001
Patients with eGFR 15–29 ml/min/1.73 m², % (*N*)	6.8 (48)	1.0 (15)	<.001
Hemoglobin level (g/dl), mean, (SD)	13.1 (1.9)	14.0 (1.4)	.016
Platelets count (×10^3^/mm^3^), mean, (SD)	226.8 (89.5)	225.1 (62.6)	.006
Serum uric acid concentration (mg/dl), mean, (SD)	6.4 (2.3)	5.7 (3.9)	.017
AspAT activity (IU/l), mean, (SD)	93.8 (179.4)	59.5 (74.6)	.049
ALAT activity (IU/l), mean, (SD)	51.2 (95.8)	41.5 (155.2)	.111
Cholesterol concentration (mg/dl), mean, (SD)	192.0 (49.2)	207.1 (48.5)	<.001
LDL concentration (mg/dl), mean, (SD)	125.8 (44.7)	137.8 (45.0)	<.001
HDL concentration (mg/dl), mean, (SD)	45.1 (13.2)	48.2 (13.2)	.155
Triglycerides concentration (mg/dl), mean, (SD)	113.5 (73.6)	122.1 (90.8)	.013
Fibrinogen concentration (mg/dl), mean, (SD)	465.3 (130)	416.2 (104)	.388

ALAT: alanine aminotransferase; AspAT: aspartate aminotransferase; BMI: body mass index; eGFR: estimated glomerular filtration rate; HDL: high-density lipoprotein; LDL: low-density lipoprotein; NSTEMI: non-ST segment elevation myocardial infarction; PC–AKI: post-contrast acute kidney injury; STEMI: ST-segment elevation myocardial infarction.

Postprocedural eGFR change rate correlates inversely with platelets count (*R* = −0.12) in STEMI patients. There is a positive correlation between the postprocedural eGFR change rate in STEMI group and AspAT (*R* = 0.14) and ALAT (*R* = 0.08). In the NSTEMI group, a positive correlation was noted between postprocedural eGFR change rate and LDL cholesterol concentration (*R* = 0.05) (Table 5).

**Table 5.** Spearman’s correlation rank between eGFR change rate and biochemical results.

Variables	All patients	NSTEMI	STEMI
eGFR using CKD-EPI	**−0.2**	**−0.2**	**−0.2**
Hemoglobin	0.01	0.01	0.02
PLT	**−**0.04	0.01	**−0.12**
Uric acid	**−**0.01	**−**0.01	**−**0.01
AspAT	**0.11**	0.04	**0.14**
ALAT	**0.05**	**−**0.02	**0.08 **
Total cholesterol	0.03	0.04	**−**0.01
LDL	**0.04**	**0.05**	0.01
HDL	0.02	0.02	**−**0.01
Triglycerides	**−**0.02	**−**0.04	**−**0.01
Fibrinogen	0.01	0.01	**−**0.01

ALAT: alanine aminotransferase; AspAT: aspartate aminotransferase; eGFR: estimated glomerular filtration rate; HDL: high-density lipoprotein; LDL: low-density lipoprotein; PLT: platelets count. The bold values represents as statistically significant *p*<.001 values.

The risk of CI-AKI increased with an increase in creatinine concentration (RR: 2.99, 95%CI: 1.567–5.721, *p* < .001) but also with the presence of diabetes mellitus (RR: 2.143, 95%CI: 1.029–4.463, *p* = .042) and atrial fibrillation (RR: 2.289, 95%CI: 1.056–4.959, *p* = .036) (Table 6).

**Table 6.** Multivariable logistic regression forward stepwise Wald method – risk ratio for risk of post-contrast acute kidney injury.

	*p*	RR	CI for RR (95%)
Creatinine concentration (for an increase of 1 mg/dl)	<.001	2.99	1.567 − 5.721
Diabetes mellitus	.042	2.143	1.029 − 4.463
Atrial fibrillation	.036	2.289	1.056 − 4.959

*R*^2^ Nagelkerke = 0.369.

Multivariable regression analysis showed that the risk of death in both STEMI and NSTEMI groups was greater with an increase in postprocedural creatinine concentration (for an increase of 1 mg/dl risk ratio was: 2.254 95%CI: 1.481–3.424, *p* < .001), with age (RR: 1.060, 95%CI: 1.048–1.072, *p* < .001), the presence of atrial fibrillation (RR: 1.695, 95%CI: 1.251–2.297, *p* = .034), and increase in fibrinogen concentration (RR: 1.028, 95%CI: 1.018–1.068, *p* < .001) but also decreased with an increase in hemoglobin concentration (RR: 0.895, 95%CI: 0.826–0.970, *p* = .007) (Tables 7–9).

**Table 7.** Multivariable logistic regression forward stepwise Wald method –risk ratio for risk of death – all population.

	*p*	RR	CI for RR (95%)
Male	.002	1.503	1.156 − 1.955
Age (for an increase of 1 year)	<.001	1.060	1.048 − 1.072
Postprocedural creatinine concentration (for an increase of 1 mg/dl)	<.001	2.254	1.481 − 3.424
Diabetes mellitus	.004	1.512	1.145 − 1.977
Atrial fibrillation	.034	1.695	1.251 − 2.297
Hemoglobin concentration (for an increase of 1 g/dl)	.007	0.895	0.826 − 0.970
Fibrinogen concentration (for an increase of 10 mg/dl)	<.001	1.028	1.018 − 1.068

*R*² Nagelkerke = 0.469.

**Table 8.** Multivariable logistic regression forward stepwise Wald method – risk of death in NSTEMI population.

	*p*	RR	CI for RR (95%)
Age (for an increase of 1 year)	.008	1.067	1.052 − 1.083
Postprocedural creatinine concentration (for an increase of 1 mg/dl)	<.001	2.542	1.281 − 3.124.
Atrial fibrillation	.010	1.689	1.132 − 2.520
Hemoglobin concentration (for an increase of 1 g/dl)	.025	0.887	0.787 − 0.986
Fibrinogen concertation (for an increase of 10 mg/dl)	<.001	1.026	1.012 − 1.040

*R*² Nagelkerke = 0.443.

**Table 9.** Multivariable logistic regression forward stepwise Wald method – risk of death in STEMI population.

	*p*	RR	CI for RR (95%)
Male sex	.007	1.787	1.176 − 2.716
Age (for an increase of 1 year)	<.001	1.047	1.029 − 1.066
BMI (for an increase of 1 kg/m^2^)	.031	0.961	0.926 − 0.998
Diabetes mellitus	<.001	2.150	1.373 − 3.369
Atrial Fibrillation	.012	1.847	1.143 − 2.986
Hemoglobin concentration (for an increase of 1 g/dl)	.047	0.883	0.781 − 0.998
Fibrinogen concentration (for an increase of 10 mg/dl)	<.001	1.032	1.015 − 1.049

*R*² Nagelkerke = 0.495.

## 4. Discussion

In recent years, the epidemiology of ACSs has changed in Poland and Europe. Non-ST-segment elevation myocardial infarction starts to play a dominant role, which is in line with our study [10,11]. Short-term mortality is higher in the STEMI group, however, in the case of long-term follow-up, the death rate for NSTEMI becomes twice as high in comparison to STEMI [10,12]. These data are reflected in our analysis. Differences in prognosis over time result from different characteristics of patients, people predisposed to NSTEMI are usually older and have more coexisting diseases, especially diabetes and renal failure.

In this investigation, CKD occurred in one out of four people and more often affected those with NSTEMI. Patients with CKD had worse outcomes regardless of the type of ACS. These results can be found in previous studies, CKD of any degree is a potential and independent risk factor for adverse outcomes. [13,14]. Population with CKD had more often comorbidities, such as hypertension, diabetes mellitus, atrial fibrillation, and advanced age which is mostly traditional risk factors for cardiovascular diseases. CKD is also associated with a high burden of cardiovascular diseases, especially coronary artery disease which has an impact on poorer long- and short-term outcomes after ACS [15–17]. These findings are reflected in current guidelines. Individuals with CKD are considered as a group of high risk for cardiovascular diseases and other adverse outcomes which supports calls for more intensive intervention in patients with CKD to prevent them. Therapeutic strategies that have been proved to prevent cardiovascular events in patients with CKD include aggressive blood pressure control, statins, and angiotensin-converting enzyme inhibitors/angiotensin receptor blockers [18].

An additional burden, especially in the group of CKD patients, was the occurrence of PC-AKI. Contrast-induced acute kidney injury occurred in 99 patients, in every 20th patient with STEMI, however, in the group of patients with CKD history almost 10 percent of the patients were affected. Contrast nephropathy is a common complication, however, the risk of death associated with it is lower than the risk of untreated infarction. PCI is the gold standard procedure [19]. Its effectiveness in reducing mortality is greater than other reperfusion therapy strategies. There are several methods of the prevention of acute renal injury after the administration of contrast, which should be used in high-risk patients (the population with CKD) [20]. However, scientific work so far has focused mainly on the risk of infarction in CKD patients or on the influence of GFR on infarct severity [21,22]. This research allows us to estimate the prognosis in post-infarction patients, after the performed intervention and assess which patients require particular attention. This is a useful marker while considering the whole population with STEMI or NSTEMI infarction.

The studies performed so far report that PC-AKI has a strong impact on an increased risk for adverse clinical outcomes in patients with ACS and increased mortality in post-procedural patients. Our study confirms this theory. During the analysis, it was noted that patients with contrast nephropathy have significantly higher mortality rates [7,23–28].

Patients who are scheduled to have a contrast-enhanced diagnostic or interventional procedure should be evaluated for risk factors of PC-AKI. Previous studies report about various initiators increasing the risk of complications, where the most important is preexisting CKD. Our analysis shows that the risk of PC-AKI is twice as high in patients with diabetes and atrial fibrillation and increases also as the baseline creatinine concentration rises.

One of the most interesting results of our study is a significant role of risk factors, which seems to be underestimated. It is well known that age, diabetes mellitus, prior ACS, time from door to balloon, tachycardia, hypotension, and cardiogenic shock are independent predictors of poor prognosis in patients with ACS. In addition, Shacham et al. have shown that the presence of severe hyperglycemia on admission without previously diagnosed diabetes was an independent risk factor for the development of AKI among STEMI patients undergoing primary PCI [29]. However, in our study, the main prognostic factor was post-procedural creatinine concentration. An increase in post-procedural creatinine concentration of 1 mg/dl in long-term observation resulted in 2.2 times higher death risk ratio. An important impact of renal function was observed in other results. The mortality of patients with contrast-induced acute kidney injury was more than two times higher in the group of CKD in comparison to the rest of the population. In the literature, the impact of CKD on long-term mortality of patients with ACSs was reported with odds ratios between 1.66 and 2.8 [30,31]. In our study, PC-AKI was observed in ninety-nine patients (4.47%). Although the occurrence rate of PC-AKI was higher in the recent studies (11.2 − 16.1%), similarly to our results it was more common in patients diagnosed with STEMI [32–34].

The reason for taking PC-AKI as a strong predictor of death is not only the observation that renal function of half of PC-AKI patients did not return to the baseline concentration but also in almost half of the group it was leading to CKD [35,36]. On the other hand, patients with PC-AKI have more severe coronary artery disease, a higher burden of traditional coronary risk factors, and complications such as diabetes mellitus, hypertension, left ventricular hypertrophy, dyslipidemia, atrial fibrillation, and congestive heart failure, what had an impact on the long-term mortality.

Our results suggest that PC-AKI has a negative impact on outcomes of patients with ACS treated with invasive procedures. More attention should be paid to the prevention and diagnosis of PC-AKI. Nevertheless, necessary PCIs should not be withheld in the fear of PC-AKI.

## 5. Limitations

Our study had several limitations. Firstly, it was a retrospective, single-center study, but covering a large population. The second limitation is a high percentage of garbage codes in mortality statistics (4–30%). Thirdly, the level of creatinine before ACS and in long-term follow-up is not included in our study. Baseline creatinine values may already reflect impairment from hemodynamic changes in the setting of acute myocardial infarction. Control assessment of creatinine level would let us assess the impact of developing CKD in PC-AKI and non-PC-AKI patients and how the concentration of creatinine returns to baseline levels.

Finally, we might have potentially underestimated the real incidence of PC-AKI, as our protocol only recommended measurement of creatinine level for 2 days after admission. In addition, a potential underestimation of the phenomenon of PC-AKI may be due to the use of historical definition and creatinine level control at 48 h instead of the commonly accepted 72-h delay. Thus, we might have missed later rises in serum creatinine.

## 6. Conclusions

Chronic kidney disease affects mostly the population with NSTEMI infarction. Contrast-induced acute kidney disease is a major complication in patients with ACS and occurs more frequently in the STEMI population. The risk of CI-AKI is increased in patients with preprocedural renal insufficiency. CI-AKI may be a prognostic marker of long-term mortality in ACS patients undergoing PCI.

More attention should be paid to the prevention and diagnosis of CI-AKI but necessary PCIs should not be withheld in fear of CI-AKI.
